# Phacoemulsification Cataract Surgery Affects the Discriminative Capacity of Iris Pattern Recognition

**DOI:** 10.1038/s41598-019-47222-4

**Published:** 2019-07-31

**Authors:** Ishan Nigam, Rohit Keshari, Mayank Vatsa, Richa Singh, Kevin Bowyer

**Affiliations:** 1 Carnegie Mellon University, Pittsburgh, USA; 2 IIIT Delhi, New Delhi, India; 3 University of Notre Dame, South Bend, USA

**Keywords:** Computer science, Scientific data

## Abstract

Cataract is a common ophthalmic disorder and the leading cause of blindness worldwide. While cataract is cured via surgical procedures, its impact on iris based biometric recognition has not been effectively studied. The key objective of this research is to assess the effect of cataract surgery on the iris texture pattern as a means of personal authentication. We prepare and release the IIITD Cataract Surgery Database (CaSD) captured from 132 cataract patients using three commercial iris sensors. A non-comparative non-randomized cohort study is performed on the iris texture patterns in CaSD and authentication performance is studied using three biometric recognition systems. Performance is lower when matching pre-operative images to post-operative images (74.69 ± 9.77%) as compared to matching pre-operative images to pre-operative images (93.42 ± 1.76%). 100% recognition performance is observed on a control-group of healthy irises from 68 subjects. Authentication performance improves if cataract affected subjects are re-enrolled in the system, though re-enrollment does not ensure performance at par with pre-operative scenarios (86.67 ± 5.64%). The results indicate that cataract surgery affects the discriminative nature of the iris texture pattern. This finding raises concerns about the reliability of iris-based biometric recognition systems in the context of subjects undergoing cataract surgery.

## Introduction

Cataract is a leading cause of blindness worldwide, accounting for more than one in three major ophthalmic disorders^1^. The World Health Organization (WHO) Vision 2020 initiative and similar programs have led to an increase in surgical interventions performed to combat this epidemic of preventable blindness. However, the prevalence of cataract as a public health issue continues to grow as the worldwide mean life expectancy rises^2^. While patients continue to benefit from improvements in cataract treatment, the management of cataract in developing countries remains an impediment towards addressing preventable blindness^3,4^. Long-term population-based studies conducted in developing countries report an acute deficiency in the visual acuity of operated cataract patients^5,6^. Complications arise due to lack of awareness - patients do not recognize the value of early treatment, have a high threshold of tolerance to medical intervention, and fail to recognize the importance of post-operative care, creating impediments to successful restoration of vision. Further, alteration of the iris texture may occur due to depigmentation, localized atrophy, tears in the iris sphincter, surgical coloboma, or other factors which may have been avoidable.

**Figure 1:**
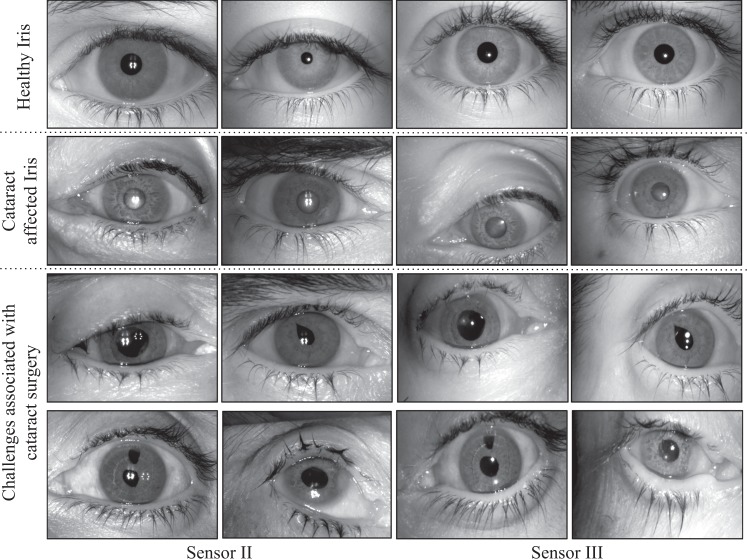
Illustrating healthy iris images and cataract affected iris images from two different sensors. The images of eyes affected by cataract are artifically dilated with Tropicacyl Plus solution to illustrate the effect of cataract on the image captured with biometric sensors. The images in the first and third colums, as well as in the second and fourth columns, show cross-sensor iris patterns captured for the same individual.

In recent years, biometric recognition methods (i.e. use of distinct physiological or behavioral characteristics to uniquely identify individuals), including the usage of iris texture patterns, are being integrated into large-scale authentication systems. The formation of iris patterns is determined by random events in the development of morphological structures in the component tissue and the resultant discriminative nature of the iris pattern serves as a reliable basis for person authentication^7^. The success of the (healthy) iris pattern as a biometric characteristic has led to the initiation of several large-scale biometric recognition systems, of which India’s Aadhaar program^8^ is the largest and best known. The use of the iris pattern as a means of large-scale authentication has also resulted in the need for understanding the effects of common ophthalmic disorders and medical procedures on identification performance. Some studies have examined the effect of eye pathology on iris recognition^9,10^. The effects of developing complications such as anterior uveitis, iritis, macular coloboma, or cataract may cause recognition systems to fail. India, home to the Aadhaar program with over 1.2 billion identities, is expected to host approximately 8,000,000 cataract patients undergoing surgery annually by 2020^11^. In 2010, more than 8 million patients in Germany and more than 80,000 patients in Austria underwent cataract surgery^12,13^. Thus, it becomes imperative that we understand the effect of cataract surgery on iris biometric recognition. Figure 1 illustrates samples of healthy images and iris images with cataract.

Methods in the literature^14–16^ have previously studied the effect of cataract surgery on the iris pattern. Roizenblatt *et al*.^14^ first studied the effect of cataract surgery on patients in a study conducted in South America and found that surgical intervention affects iris biometric recognition. Dhir *et al*.^15^ carried out a preliminary study in Europe, suggesting that cataract surgery does not necessarily affect iris biometric recognition. Recently, Seyeddain *et al*.^16^ conducted a study which concluded that iris biometric recognition may be affected by cataract surgery under certain circumstances. Table 1 summarizes the approaches in the literature along with a brief overview of the proposed study. These studies do not point towards a consensus on the effect of cataract surgery on iris biometric recognition. Moreover, the varying geographical locations and associated socio-economic conditions for these studies indicate that the medical procedures, and thus the medical outcomes, may not be uniform^17^.

**Table 1:** Comparative analysis of studies on the effect of cataract surgery.

Parameters	Roizenblatt *et al*.^14^	Dhir *et al*.^15^	Seyeddain *et al*.^16^	Proposed Study
Total subjects	55	15	173	132
Single-sensor data	55	15	173	83
Cross-sensor data	0	0	0	59
Time from surgery to post-op imaging	1 month	2 weeks	2–24 hours	2–8 days
Number of iris sensors	1	1	1	3
Number of recognition systems	1	1	1	2
Location	South America	Europe	Europe	Asia

In a departure from prior methods, we perform cross-sensor iris recognition using multiple pattern matching algorithms. The cross-sensor study performed with multiple matchers allows us to objectively investigate whether the iris pattern changes due to surgical intervention for cataract.

Large scale biometric recognition systems based on iris pattern recognition require an understanding of how underlying covariates may affect the iris pattern. In this paper, we study the recognition performance of several commercial iris biometric recognition systems to gain a better understanding of how surgical intervention for cataract may affect iris recognition performance in large-scale biometric recognition systems.

## Results

Iris pattern recognition in non-ideal scenarios is susceptible to errors during segmentation of the iris pattern and while performing matching. Our findings on the effect of cataract surgery on segmentation performance as well as matching performance of iris biometric recognition are categorically reported below.

### Segmentation analysis

**Figure 2:**
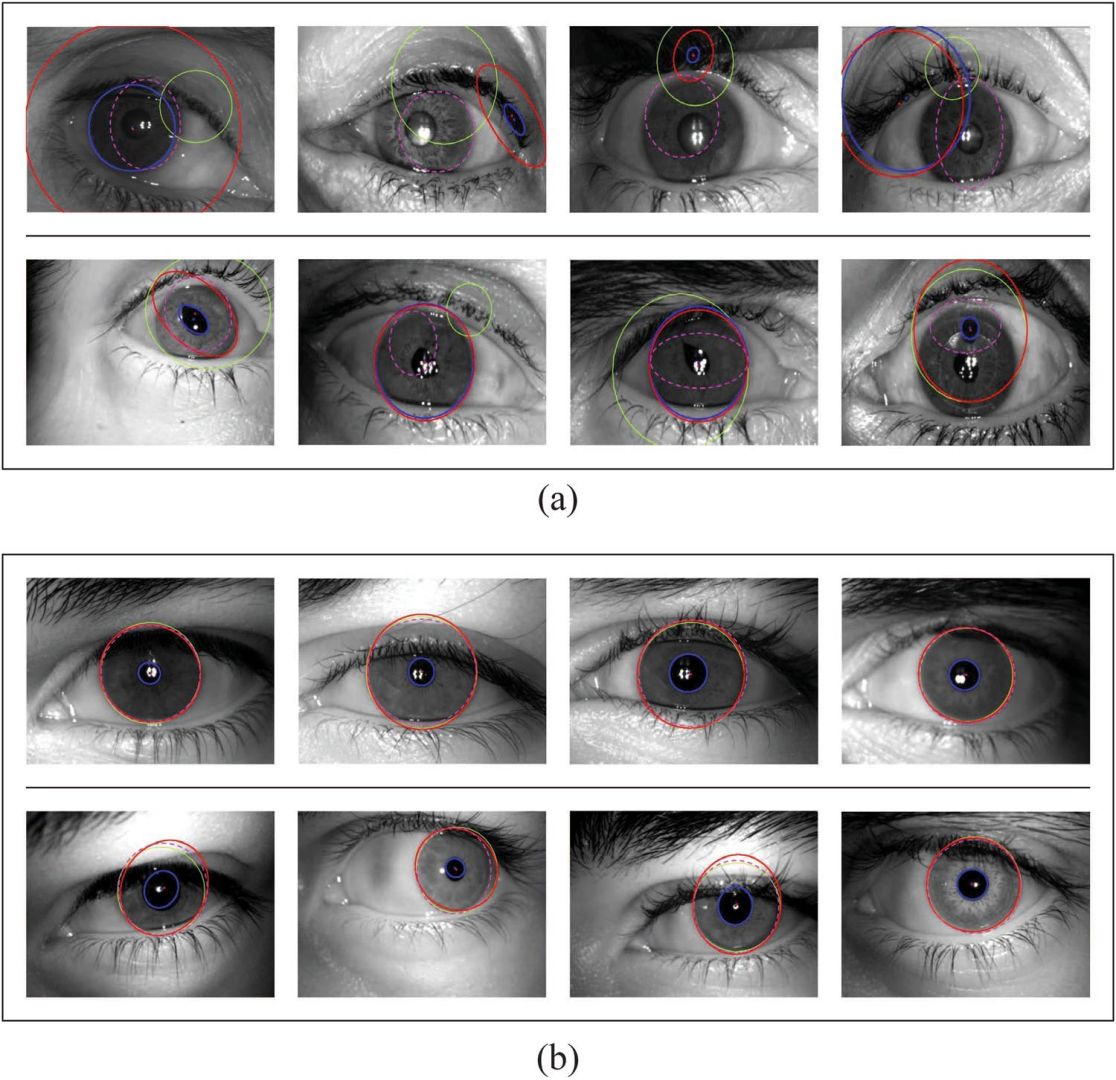
(**a**) Failure of segmentation for subjects in the IIITD Cataract Surgery Database. The first row shows pre-surgery segmentation failures, and the second row shows post-surgery segmentation failures. (**b**) Successful segmentation of healthy irises using the same matchers. Green: Matcher I, magenta: Matcher II, blue and red: Vatsa *et al*.^18^.

Segmentation of the iris pattern images is studied using the algorithm developed by Vatsa *et al*.^18^. Visual analysis of the segmentation failure cases using Matcher I and Matcher II (described in the Materials and Methods section) is also performed by the authors. It is observed that errors in segmentation of the iris pattern occur before as well as after cataract surgery. Figure 2(a) illustrates exemplars of incorrectly segmented iris patterns, and Figure 2(b) shows successful segmentation attempts for healthy irises captured using the same near infrared sensors.

Errors in segmentation prior to surgery are primarily due to whitening of the pupil, which affects the automated iris pattern segmentation algorithms; this error may be corrected after surgical intervention. The leading causes for incorrect segmentation of the iris after surgical intervention are multiple specular reflections of the near-infrared light source from the intra-ocular lens implant, punctures in the iris pattern, and complications resulting from the surgical procedure. The specular reflection pixel count detected in pre-surgery images for Sensor II and Sensor III is 508.5 ± 312.1 and 148.0 ± 80.6, respectively. Specular reflection pixel count in post-surgery images for Sensor II and Sensor III is 636.5 ± 320.3 and 239 ± 151.9, respectively. Specular reflection is also studied for a similar number of healthy iris images as a control group; the specular reflection count in healthy iris images for Sensor II and Sensor III is 451.5 ± 158.2 and 145 ± 153.82, respectively. We also visually inspect the number of iris samples which fail to be processed due to cataract, specular reflection, or morphological changes. Table 2 summarizes the extent of these failures before surgical intervention (Pre-surgery) and after surgical intervention (Post-surgery). In these cases, the segmentation algorithms failed to provide the region of interest for feature extraction and matching. With healthy control group images, none of the images failed to segment iris. These results show that the cataract affects iris segmentation step.

**Table 2:** Number of iris samples which failed to be processed, before and after surgical intervention.

Sensor	Pre-surgery		Post-surgery		
	Cataract	Specular Reflection	Specular Reflection	Morphological Change	Morphological Change & Specular Reflection
Sensor I	4	1	5	0	2
Sensor II	8	0	6	8	9
Sensor III	0	0	1	1	2

The incidence of specular reflection acutely increases for post-surgery iris samples.

### Matching analysis

We study the matching performance of Matcher I and Matcher II on data collected using the three sensors. The Genuine Accept Rate (GAR) for the systems at 0% False Accept Rate (FAR) is used to compare matching performance for the iris patterns. 49 unique irises are imaged using Sensor I. Iris pattern data collected using Sensor II consists of iris patterns of 74 cataract-affected eyes. 68 unique irises are imaged using Sensor III.

The drop in GAR at 0% FAR for Matcher I applied to Sensor I data is 29.96%. The drop in GAR at 0% FAR for Matcher I applied to Sensor II data and Sensor III data is 6.01% and 6.04%, respectively. The cross-sensor experiment performed on a subset of Sensor II and Sensor III data is observed to have a 11.85% drop in the recognition performance.

**Figure 3:**
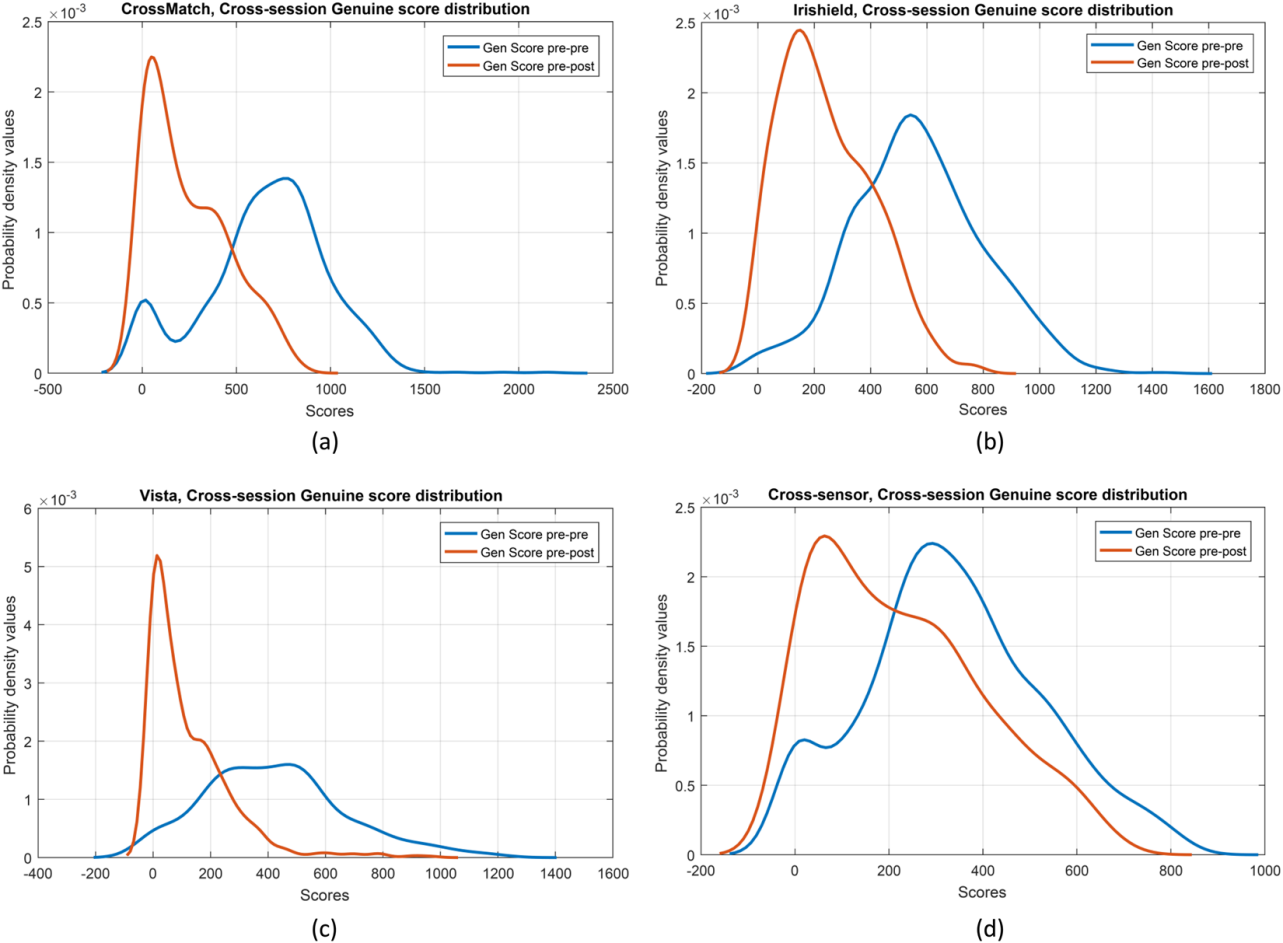
Pre-surgery and post-surgery genuine score distributions of different sensors using a commercial matcher. The first three figures correspond to same sensor matching and the last figure corresponds to cross-sensor matching.

Matching performance for Matcher II follows similar trends. The drop in GAR at 0% FAR for Matcher II applied to Sensor I data is 36.4%. The drop in GAR at 0% FAR for Matcher II applied to Sensor II and Sensor III data is 21.03% and 13.14%, respectively. The cross-sensor experiment performed on a subset of Sensor II and Sensor III data is observed to have a 13.24% drop in recognition performance. Table 3 elaborates upon the results for iris biometric matching for Matcher I and Matcher II. Figure 3 shows the score distribution from one of the commercial matchers which illustrates the shift in genuine match score distribution.

**Table 3:** Matcher I and Matcher II - Genuine Accept Rate (GAR) at 0% False Accept Rate (FAR) for the IIITD Cataract Surgery Database.

	Experiment	Subjects	Pre-Pre (%)	Post-Post (%)	Pre-Post (%)	Healthy Iris (Control) (%)
Matcher I	Sensor I	49	94.29	78.37	64.33	100.00
	Sensor II	74	91.20	85.16	75.19	96.11
	Sensor III	68	94.99	96.21	88.98	96.92
	Cross-Sensor	59	91.04	88.09	79.19	95.10
Matcher II	Sensor I	49	95.07	84.01	58.67	100.00
	Sensor II	74	91.89	85.70	70.86	98.44
	Sensor III	68	94.85	92.77	81.71	99.19
	Cross-Sensor	59	91.84	83.05	78.60	98.55

Columns 4, 5, 6 respectively represent GAR for matching pre-surgery iris samples to pre-surgery samples, post-surgery samples to post-surgery samples, and pre-surgery samples to post-surgery samples. Column 7 represents matching of healthy iris samples collected from a similar population demographic.

A control group of 68 healthy irises is used for studying matching performance using same recognition algorithm. The GAR at 0% FAR is not affected at all when recognition is performed using both Matcher I and Matcher II. The null hypothesis (*H*_0_) of the t-test we perform states that the two experiments will yield genuine scores sampled from the same distribution. The alternate hypothesis (*H*_1_) of the t-test states that the distribution of genuine scores obtained from the two experiments will not correspond to the same distribution. The null hypothesis of the t-test fails for Sensor I with *p* = 1.18 × 10^−166^, fails for Sensor II with *p* = 7.49 × 10^−180^, and fails for Sensor III with *p* = 2.60 × 10^−111^. The null hypothesis for the t-test applied to the cross-sensor experiment also fails with *p* = 4.10 × 10^−37^.

## Discussion

We perform a non-comparative cohort study to study how cataract and cataract surgery affects iris-based authentication. The IIITD Cataract Surgery Database is the largest publicly-available database of iris images collected from patients with cataract. It was prepared to study iris authentication in unconstrained scenarios mirroring real-world applications. We study the iris patterns of 132 cataract surgery patients using three iris sensors. Two commercial iris biometric matchers are used to study the effect of cataract surgery on the robustness of matching performance of the iris pattern as a biometric characteristic. Earlier studies have established that inter-session variations and cross-sensor variations have little effect on iris pattern recognition^19^, thus, not requiring a comparative cohort study analysis.

Previous research papers on understanding the effect of cataract surgery on iris biometric recognition involved studying patients using a single iris pattern imaging sensor in a constrained clinical environment. It is suggested that cataract surgical interventions do not affect the recognition performance of iris pattern biometric authentication systems^15^. However, this conclusion is based on a study of only fifteen patients, which may limit the generality of its conclusions. The same study also indicates that all surgical complication cases are treated as part of their exclusion criteria.

A more recent study was conducted involving 176 unique irises in Austria, which suggested that standard cataract surgery is not a limiting factor for iris recognition in the large majority of cases^16^. However, the challenges associated with cataract surgery performed in developing regions are not considered in this study. The socio-economic condition of patients in developing regions (such as South America and Asia) result in poor visual acuity following surgery due to a lack of proactive attention to non-fatal medical conditions. Cabrera *et al*.^20^, recently conducted a study in Mexico to determine socio-economic factors associated with cataract patients; more than half the patients had not been educated beyond the primary level, while half the patients enrolled in primary ophthalmological care an year after the onset of symptoms. Such factors result in misinformation and lack of understanding of a treatable condition as well as lack of attention to post-operative care. Similarly, other studies have also shown that outcomes of cataract surgery performed in rural parts were sub-optimal^21–23^. Thus, an average cataract patient in a developing country is more likely to be affected by surgical complications as well as post-treatment complications than a patient in a developed country with access to quality medical care.

While Roizenblatt *et al*.^14^ choose a period of one month to perform post-operative matching to ensure that healing and chronic tissue retraction are complete, we follow a more realistic time period (5 ± 2.5 days) in which major visual signs of ocular surgery have subsided and the patient is mobile and ready to move around and interact with iris biometric systems. Analysis of the segmentation of iris patterns indicates that advanced cataract, whether untreated or treated, may result in failure of state-of-art iris pattern segmentation algorithms. As shown in Figure 1, high intensity spots are observed in the near-infrared iris pattern images collected after surgical intervention, resulting from reflections off the surface of the intraocular lens implant. These spots are not observed before surgery and interfere with automated segmentation of the iris pattern in post-surgery images, thus, deteriorating iris pattern recognition performance.

**Figure 4:**
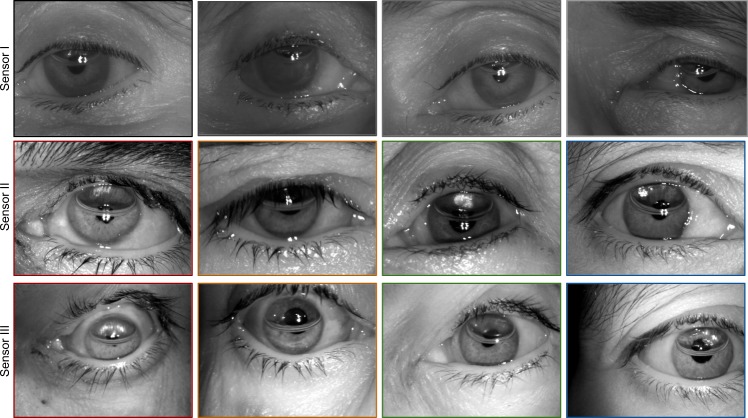
Post cataract surgery samples collected after one day of surgery.

Seyeddain *et al*.^16^ capture post-operative images 2–24 hours after surgical intervention. Deterioration in the performance of the iris pattern for comparison of pre-operative images to post-operative images is attributed to epithelial edema and Descemet folds. The matching performance of the iris pattern is observed to drop for single sensor as well as cross-sensor matching in our study as well, though the period of capture for post-operative images in our study is 2–8 days. We also visually analyze post-operative iris images captured 24 hours after surgery and, as shown in Figure 4, they are observed to be unfit for iris recognition due to immediate short-term post-operative artifacts. In all these cases, all the images fail to segment and match; therefore, we assert that iris recognition should not be performed within 24 hours of cataract surgery.

Iris segmentation algorithms rely on the elliptical pupil boundary to perform iris segmentation. Specular reflection of the eye pupil has the potential to confound boundary detection algorithms. Errors in boundary detection may result in degradations in the segmentation of the iris, lowering iris recognition performance. Specular reflection on iris images poses a challenge to automated iris pattern segmentation. It is observed that the whitening of the pupil in pre-surgery iris images results in lower specular reflection on the pupil compared to healthy iris images. However, we observe higher specular reflection in post-surgery images, which may be attributed to the intra-ocular lens implant placed during the phacoemulsification procedure. This increase in specular reflection leads to problems in automated iris pattern segmentation.

Based on assertions from previous studies^15,16^, it is expected that the match scores for pre-operative images compared to pre-operative images and pre-operative images compared to post-operative images correspond to the same score distribution. However, this hypothesis is found to fail for the match score distributions of genuine comparisons of iris pattern data from all three sensors, as well as the data captured using both Sensor II and Sensor III. As iris image biometric recognition becomes a common means of identifying individuals, it will be critical that the ophthalmological community improves upon the practical aspects associated with phacoemulsification-based cataract surgical intervention to aid iris biometric recognition. In developing countries including India, sub-optimal visual outcomes have been observed in surgically treated individuals in the past^21–24^. However, the phacoemulsification method performed by an experienced cataract surgeon with implantation of foldable as well as rigid intra-ocular lens is reported to give satisfactory results^25^.

Our findings suggest that the effects of cataract surgery on iris-based biometric authentication may not have been correctly and completely understood in the past. Comparing the performance of matching pre-surgery images to pre-surgery images with the performance of matching pre-surgery images to post-surgery images, we find a statistically significant reduction in performance for matching pre-surgery images to post-surgery images. This indicates that cataract surgery using the phacoemulsification method induces a change in biometric imaging of the iris pattern that impedes current iris segmentation and matching algorithms. In principle, we recommend two methods to mitigate the effects of cataract surgery on iris pattern recognition. First, we believe that the problem may be addressed to a limited extent within the current setup by re-enrolling cataract patients after surgical intervention. Since the matching performance of post-surgery images to post-surgery images is quite high, re-enrollment is likely to limit the effects of cataract surgery. This is especially true for the performance of Sensor III for both Matcher I and Matcher II; Sensor III is a mobile iris image sensor which is characteristic of sensors used in several biometric programs. Second, the results obtained from Sensor III indicate that iris recognition sensors should be designed to reduce specular reflection. Moreover, newer algorithms should be developed that perform automated iris recognition cognizant of the artifacts introduced by cataract surgery in the imaging of iris texture patterns. In conclusion, this study suggests that it is important for automated iris recognition systems to take into account the effect of cataract surgery, and possibly other surgical interventions performed on the eye, to ensure that the iris texture pattern remains a robust biometric characteristic.

We qualitatively observe an increase in the overall time required to capture data from the patients, which was primarily due to the increased specular reflection as well as morphological changes in the pattern. In the future, we plan to conduct a longitudinal study in order to make practical (data capture level) recommendations towards large scale iris recognition projects. Further, we plan to conduct a follow-up study to quantitatively determine the post-operative refractory time period during which the iris texture pattern is not reliable for large-scale authentication systems.

## Materials and Methods

We study the effect of cataract surgery on the performance of the iris texture pattern as a means for identifying individuals. Iris pattern data was collected and studied in cataract patients before and after surgical intervention was performed via the phacoemulsification method. Written informed consent was obtained from all participants to study and distribute their iris images in an anonymized fashion for non-commercial research purposes.

### IIITD cataract surgery database

Early studies^14,15^ in the literature focused on a small set of patient data, while recently Seyeddain *et al*.^16^ described a single-sensor study conducted within twenty-four hours of the surgical procedure. The IIITD Cataract Surgery Database (CaSD), collected during the period 2012 to 2015, is prepared to investigate the challenges of iris pattern recognition in large-scale biometric recognition systems. Iris pattern data from 132 individuals is collected by the Image Analysis and Biometrics Lab at IIIT Delhi, India. 64 left eyes (48.48%) and 68 right eyes (51.52%) are included in the study. 73 patients (55.30%) are males and 59 patients (44.70%) are females. Approval is obtained from the IIIT Delhi Ethics Committee for collection of the data utilized in the study and consent to participate in this study is obtained from each participant. All research is performed in accordance with the relevant guidelines and regulations outlined in the Declaration of Helsinki^26^. The database is available to the researchers for non-profit purposes at http://www.iab-rubric.org/resources.html.

The data, collected at two different locations in North India, is referred to as CaSD Set I and CaSD Set II, in our study. Subjects in both the sets have the same ethnic background. In all the cases, pre-surgery images are captured just before the cataract surgery and post-operative images are captured 2–8 days after the surgery. Further, visual acuity in pre-operative images for CaSD is 0.3 logMAR, and visual acuity improves to 0.1 logMAR for post-operative images. We ensure that no patient overlap occurs for Set I and Set II iris image data in CaSD. While CaSD Set I only consists of data collected using one biometric sensor, a subset of the data in CaSD Set II is collected using two iris biometric sensors. The CaSD data is collected controlling for any changes in the physical environment; no additional illumination source is used, except the inbuilt near-infrared (NIR) LEDs in the sensors. Individuals are inducted in the study after a certified medical professional establishes that the patient suffered from cataract and is suitable to undergo surgical intervention. Tropicacyl Plus (generic name: tropicamide ophthalmic), used to relax eye muscles, is administered during the preliminary pre-operative examination as well as the post-operative follow-up examination. However, iris images are captured a few hours after administering the medicine. Finally, the phacoemulsification method of surgical intervention is used to introduce a PMMA intra-ocular lens implant to replace a cataract affected eye lens.

CaSD Set I consists of iris image data collected from 49 patients using the Vista Imaging VistaFA2E sensor (Sensor I with 6 inbuilt NIR LEDs). CaSD Set II consists of iris image data collected using two sensors. 74 patients are studied using the Crossmatch I Scan sensor (Sensor II with 6 inbuilt NIR LEDs) and 68 patients are studied using the IriTech IriShield sensor (Sensor III with single inbuilt NIR LED). Sensor II is used in large-scale iris recognition systems for enrollment and Sensor III is a mobile iris sensor representing the challenges of unconstrained iris recognition. CaSD Set IIA consists of single-sensor iris data collected from 83 patients. CaSD Set IIB consists of cross-sensor iris data collected from 59 patients using both Sensor II and Sensor III. Table 4 describes the characteristics of the database.

**Table 4:** Characteristics of the IIITD Cataract Surgery Database.

	CaSD Set I	CaSD Set IIA	CaSD Set IIB
Single-sensor data subjects	49	83	—
Cross-sensor data subjects	—	—	59
Number of sessions	2	2	2
Samples per session	4	4	8
Total Samples	392	664	944

The exclusion criteria for the study comprises of patients requiring ocular procedures such as corneal grafts, glaucoma treatments (both laser-operated as well as topical medicinally treated), uveitis, diabetic retinopathy, and vitreous detachment. We also exclude patients with intra-operative complications or post-operative complications, as well as patients who refused to provide consent towards distribution of iris biometric data.

### Methods used for analysis

#### Specular Reflection Analysis

The effect of specular reflection is studied for pre-surgery images, post-surgery images, as well as a set of healthy iris images as a control group. Specular reflection is detected using a method popular in literature^27^: hard thresholding is performed on the iris image to populate a binary image mask with regions corresponding to high intensity values. Morphological search operations are performed on the image mask to localize regions affected by specular reflection. The metric used to express the extent of specular reflection in the iris image is the number of pixels in the segmented iris and pupil regions that have high intensity values. The intensity thresholds and morphological operations used to perform the above computations comply with methods prevalent in the iris biometric literature.

#### Iris Pattern Matching Analysis

The matching performance of iris pattern images is analyzed using two commercial Software Development Kits (SDK), which represent the state-of-art in iris biometrics. The first recognition system is the Neurotechnology VeriEye Commercial SDK (Matcher I). The second matcher used in our study remains anonymous due to a license agreement with the sensor manufacturer for academic research purposes (Matcher II). We study the matching performance of data collected individually using the three sensors. Additionally, a cross-sensor experiment is performed on CaSD Set IIB to study challenges associated with biometric recognition performed in large-scale systems such as the Aadhaar program, which employs multiple sensors for enrollment and authentication. In order to analyze the results better, an academic algorithm developed by Vatsa *et al*.^18^ is used which is based on active contours. This segmentation algorithm is also used while performing specular reflection analysis.

A three-fold analysis is performed for each experiment - matching images captured before surgery to images captured before surgery (*Pre-Pre matching*), matching images captured before surgery to images captured after surgery (*Pre-Post matching*), and matching images captured after surgery to images captured after surgery (*Post-Post matching*). Matching results are showcased with Genuine Accept Rate (GAR) (or True Accept Rate) with fixed 0% False Accept Rate (FAR).

We also study the statistical significance of the scores obtained from Pre-Pre matching compared to Pre-Post matching to understand whether there is a change in the population of scores obtained during these comparisons. A paired t-test is performed to evaluate the statistical significance of the genuine matches from the two experiments. The paired t-test is evaluated at 0.01 significance level for the following null hypothesis and alternate hypothesis, respectively:

$$\begin{array}{rcl}{H}_{0}:{\mu }_{pre\mbox{--}pre} & = & {\mu }_{pre\mbox{--}post}\\ {H}_{1}:{\mu }_{pre\mbox{--}pre} & \ne  & {\mu }_{pre\mbox{--}post}\end{array}$$

where, *μ*_*pre*–*pre*_ represents the mean score for matching pre-surgery images to pre-surgery images, and *μ*_*pre*–*post*_ represents the mean score for matching pre-surgery images to post-surgery images.
